# A strategic framework for managing gestational diabetes in Mexico

**DOI:** 10.1186/s41256-025-00406-0

**Published:** 2025-03-01

**Authors:** Luis Alberto Martinez-Juarez, Héctor Gallardo-Rincón, Rodrigo Saucedo-Martínez, Ricardo Mújica-Rosales, Enrique Reyes-Muñoz, Diego-Abelardo Álvarez-Hernández, Roberto Tapia-Conyer

**Affiliations:** 1 Johns Hopkins Center for Humanitarian Health, 615 N. Wolfe Street, Baltimore, MD 21205 USA; 2 Humanitarian and Conflict Response Institute, University of Manchester, Ellen Wilkinson Building, Manchester, M15 6JA UK; 3 Health Sciences University Center, University of Guadalajara, Sierra Mojada 950, Independencia Oriente, 44340 Guadalajara, Jalisco Mexico; 4 Carlos Slim Foundation, Lago Zurich 245, Presa Falcon Building (Floor 20), Miguel Hidalgo, 11529 Mexico City, Mexico; 5 National Institute of Perinatology Isidro Espinosa de los Reyes, Montes Urales 800. Col. Lomas de Virreyes, 11000 Miguel Hidalgo, Mexico City, Mexico; 6 National Autonomous University of Mexico, 04510 Coyoacán, Mexico City, Mexico

**Keywords:** Gestational diabetes, Mexico, Health policy interventions, Prenatal care, Public health strategy, Diabetes management, Health equity, Early detection, Healthcare access, Policy brief

## Abstract

Gestational Diabetes (GDM) is a prevalent health challenge in Mexico, affecting 10–14% of pregnancies but detected in only about 5.1% of cases, highlighting a critical gap in the healthcare system. This underdiagnosis poses severe health risks to mothers and children and reflects broader systemic healthcare failures. The disparity in detection rates points to insufficient screening protocols and uneven access to care, particularly affecting rural areas. Additionally, a lack of integrated digital health solutions exacerbates these issues, leading to inconsistent management and follow-up of diagnosed cases. The current reactive healthcare policies fail to prioritize early intervention and comprehensive patient education, crucial for effective GDM management. This paper calls for immediate and coordinated policy action to standardize GDM screening using updated protocols across all healthcare settings, bolster digital health infrastructure for better surveillance and management, and launch an extensive public health campaign focused on GDM awareness and education. These measures should be rigorously evaluated and adapted based on ongoing research and feedback to ensure they meet the needs of all segments of the population. Addressing these challenges head-on will improve health outcomes for mothers and children and reduce long-term healthcare costs associated with GDM complications.

## Introduction

In Mexico, the management of Gestational Diabetes (GDM) confronts profound systemic challenges, mirroring the broader issues within the national healthcare framework. As a critical public health issue, GDM affects approximately 10–14% of all pregnancies in the country [1, 2]. However, the official detection rates fall significantly short of these figures, pointing to substantial gaps in screening and early diagnosis [3]. The data from the Dynamic Cubes of the DGIS (Directorate General of Health Information) reveals that in 2022, only 38,555 diagnoses of any type of diabetes were registered among 746,524 pregnant women attended by the Ministry of Health. This equates to a detection rate of only 5.1% [3], starkly contrasting with the expected prevalence of 12–14% highlighted by studies such as the *Cuido mi Embarazo* cohort study [1, 2]. This discrepancy not only underscores an acute underdiagnosis but also indicates a widespread failure in effectively covering and managing GDM at the national level.

This underdiagnosis and inconsistent management of GDM carry significant implications. Without appropriate screening and timely intervention, GDM escalates risks of severe health issues such as preeclampsia, cesarean deliveries, and the future development of Type 2 diabetes and cardiovascular diseases among mothers [4–6]. For newborns, it increases the risk of preterm birth, obesity, and diabetes later in life. The economic strain is equally concerning. In Mexico, the short-term health and economic burden of GDM is significant, with estimates suggesting that a pregnancy complicated by GDM incurs additional costs of approximately USD 2934.9 [7]. The total economic burden of GDM in Mexico ranges from USD 86.8 to USD 827.4 million per year, including direct medical costs such as hospitalizations and indirect costs like lost productivity [7]. This highlights the substantial financial impact on both families and the healthcare system.

To effectively address GDM in Mexico, it is crucial to consider the country's recent healthcare reforms and ongoing challenges. The dismantling of Seguro Popular and the creation of IMSS-Bienestar in 2022 marked a significant shift in how healthcare is provided to the uninsured population. IMSS-Bienestar aims to centralize healthcare services, offering free medical care and medications to individuals across 23 of 32 states. This reform has federalized state health systems into a cohesive structure under IMSS-Bienestar [8, 9]. These organizational changes should consider that disparities in healthcare access persist, particularly between urban and rural areas, exacerbated by the fragmented nature of the system and that could continue in the current transition. The variability in access results in unequal implementation of national and international guidelines for GDM screening and treatment [10]. To address these challenges, it is important to consider a multi-faceted approach that includes the development of a centralized digital health information system to streamline data collection, storage, and analysis. This system should integrate with existing health records and utilize mobile health technologies to capture data from remote areas, ensuring interoperability between different systems and enhancing real-time data accuracy and accessibility. Additionally, the successful implementation of these digital tools requires comprehensive training programs for healthcare workers on data management and the use of electronic health records (EHR). This training is essential to maximize the benefits of the new system and ensure consistent and high-quality care across all regions. By leveraging the existing infrastructure and operational plans of IMSS-Bienestar, this strategy can realistically support the management of GDM across Mexico and other conditions. These measures will not only improve health outcomes for mothers and children but also create a more equitable and efficient healthcare system that aligns with Mexico's recent structural changes [10].

## GDM management in Mexico

A structured approach, such as the framework proposed by the World Health Organization (WHO), is needed to address these challenges and ensure a more systematic and effective response. The approach towards GDM management in Mexico has been reactive rather than proactive. Current health policies inadequately address the need for universal and timely screening, which is crucial for preventing the development of severe complications. The healthcare system’s reliance on sporadic data collection methods and the absence of comprehensive tracking and reporting mechanisms exacerbate the problem, leaving significant gaps in epidemiological surveillance and public health planning. The following table outlines strategic policy recommendations based on the WHO’s Six Building Blocks framework (Table 1).

**Table 1:** Strategic policy recommendations for GDM management aligned with WHO’s Six Building Blocks of Health Systems

WHO building blocks	Revised policy recommendations
Service delivery	Ensure adherence to globally recognized screening algorithms, such as those proposed by the ADA and national guidelines, across all healthcare facilities Standardize and enforce GDM screening for all pregnant women, ensuring it is done at the recommended gestational age (24–28 weeks) using the 75g oral glucose tolerance test (75g-OGTT) Consider simplified OGTT with capillary glucose where the gold standard test is not feasible Promote risk-based assessment for GDM at the first prenatal visit, stratifying patients into low, intermediate, and high-risk groups to facilitate more efficient use of resources Promote early and regular GDM screenings, especially for high-risk women, as part of comprehensive prenatal care Develop and implement community-based interventions promoting healthier lifestyles, focusing on regular physical activity and balanced diets to prevent GDM Reinforce policies that ensure reclassification of GDM patients 6–12 weeks postpartum using the 75g-OGTT and provide annual monitoring with fasting glucose and/or HbA1c tests Strengthen postnatal care services for women with a history of GDM, including ongoing diabetes screening, counseling on the risk of developing Type 2 diabetes (DM2), and guidance on maintaining a healthy lifestyle post-pregnancy
Health Workforce	Provide regular training and education for healthcare providers on the latest GDM diagnostic and management guidelines Foster an integrated care approach involving endocrinologists, obstetricians, dietitians, and other relevant health professionals in the management of GDM Establish continuous professional development programs to keep healthcare providers updated on new research and practices Implement programs that provide nutritional and lifestyle counseling to pregnant women at risk of developing GDM from the first prenatal visit
Health Information Systems	Develop a centralized and accessible digital health information system to streamline data collection, storage, and analysis, integrating with existing health records and utilizing mobile health technologies for remote data capture Create a comprehensive and accessible nominal registry of GDM cases to help track its incidence, treatment, and outcomes Ensure interoperability between health systems and train healthcare workers on data management to maximize the benefits of these digital tools Implement routine data audits and feedback mechanisms to improve data quality and use in decision-making
Access to Essential Medicines	Guarantee the availability of essential medicines and supplies required for GDM management, including insulin and home glucose monitoring, particularly in underserved areas Utilize telemedicine services to monitor and manage women with GDM, ensuring consistent care access in rural and underserved regions Develop partnerships with pharmaceutical companies to ensure affordable access to medications and supplies Expand digital health initiatives for proactive identification and management of GDM
Financing	Secure funding to support the implementation of GDM management programs, covering training, digital health systems, and access to medications Advocate for increased budget allocation for maternal and child health services, with a focus on GDM Explore public–private partnerships to enhance resource availability and distribution across the healthcare system Ensure sustainable financial resources for long-term GDM management strategies
Leadership and Governance	Establish a mechanism for the regular review and update of national guidelines and policies on GDM based on emerging scientific evidence and global best practices Promote inter-institutional collaboration to share knowledge, experiences, and resources for better GDM management, potentially combining databases or resources to enhance GDM tracking and treatment outcomes Develop clear accountability and monitoring frameworks to ensure the effective implementation of GDM policies and programs Strengthen governance structures to support the standardized implementation of GDM screening and management protocols across all healthcare settings Support research into the pathophysiology of GDM, including studies into the genetic and epigenetic factors influencing its occurrence, to facilitate the development of novel diagnostic and therapeutic strategies Increase public awareness about GDM, its risk factors, implications, and the importance of early detection and management through health education programs Design and implement community-based interventions to promote healthier lifestyles, such as regular physical activity and a balanced diet, which are critical in preventing GDM

Moreover, there is an urgent need for enhanced patient education and engagement strategies. The existing efforts to inform and educate pregnant women about GDM are fragmented and often ineffective, failing to reach a significant portion of the population at risk. This lack of awareness and understanding among expectant mothers about GDM further impedes early detection and effective management of the condition. To bridge this gap, we propose culturally and linguistically tailored community-based education programs leveraging local health workers and utilize mobile health technologies for broader reach.

Addressing these challenges requires a multifaceted approach that includes the standardization of screening practices to ensure that every pregnant woman receives timely and adequate testing. Effective public awareness campaigns require robust infrastructure support, including expanded internet coverage, access to digital devices, and reliable mail services. Collaboration with telecommunication companies and local governments can bridge the digital divide, ensuring that all women have access to necessary resources and information. Additionally, leveraging digital health technologies could play a crucial role in bridging the gaps in data collection and patient monitoring, offering a more cohesive and real-time approach to managing GDM. Examples include the use of mobile health apps for remote glucose monitoring, telemedicine platforms for virtual consultations, and EHR for integrated patient data. Successful implementations like Mexico's *MIDO Embarazo* program, which uses digital tools to identify and manage pregnancy risks, demonstrate the potential of these technologies. Expanding these initiatives can improve accessibility and continuity of care, especially in remote areas [1, 2].

The national healthcare policy must also prioritize the training and equipping of healthcare providers across all regions with the necessary tools and knowledge to manage GDM effectively. This strategy should be complemented by a robust public health campaign aimed at raising awareness and understanding of GDM, its risks, and the critical importance of early screening and treatment. The current state of GDM management in Mexico calls for urgent, coordinated action to bridge gaps in care and information, enhance the efficiency of healthcare delivery, and ultimately improve maternal and neonatal health outcomes. Implementing these strategies requires not only commitment from healthcare authorities but also collaboration across various sectors to ensure a sustained and effective response to this escalating health challenge.

## Strategic policy recommendations

Given the high prevalence of GDM and the potential lifelong ramifications for both mother and child, developing comprehensive, effective health policies to address this issue is critical. The available data suggests that GDM is a significant health burden in Mexico, with an intricate interplay between genetic, environmental, and lifestyle factors, warranting focused interventions at the policy level.

It is recommended that national health policy should strengthen the screening, diagnosis, and management of GDM.

- First, the adherence to globally recognized screening algorithms, such as the one proposed by the American Diabetes Association (ADA) and national guidelines [11], should be ensured across all health care facilities. Importantly, efforts should be undertaken to customize and standardize these protocols to account for national health infrastructure realities and patient needs.
- Second, policies should promote more comprehensive prenatal care that includes early and regular GDM screenings, especially for high-risk women. Early prevention and appropriate treatment can help to mitigate the adverse outcomes associated with GDM for both mother and child.
- Third, considering the emergence of distinct GDM subtypes, a push towards precision medicine in GDM management should be advocated. This approach involves considering an individual's genetic, lifestyle, and environmental risk factors, paving the way for personalized treatment regimens and improved patient outcomes.
- Fourth, as the postpartum period is crucial in the ongoing management of GDM, policies should also emphasize postpartum reclassification and regular follow-up screenings for women with a GDM history. This would facilitate early detection and management of potential glucose intolerance or, thereby safeguarding the long-term health of these women.
- Fifth, national health policies should prioritize health education initiatives for pregnant women, highlighting the risk factors, potential short- and long-term complications, and management strategies for GDM. This would empower individuals to make informed decisions about their health, ultimately fostering improved patient adherence to GDM management plans.

To ensure the coherence and strategic integration of policy recommendations for addressing GDM in Mexico, it is beneficial to align them within the framework of the WHO's six building blocks model [12]. This structured approach guarantees a comprehensive and systematic strategy, effectively covering all critical aspects of GDM management. By leveraging this model, the proposed interventions can be more seamlessly implemented, monitored, and evaluated, ensuring their efficiency and effectiveness in improving health outcomes for mothers and children. Additionally, this approach provides a framework that can be understood and followed in other contexts outside of Mexico, promoting global consistency in GDM management practices.

## Data Availability

Not applicable.

## Abbreviations

EHR: Electronic health records
GDM: Gestational diabetes
DGIS: Health Information Directorate (Dirección General de Información en Salud)
ADA: American Diabetes Association
OGTT: Oral glucose tolerance test
WHO: World Health Organization
